# Do psychosocial factors affect the (occupational) well-being of German police officers? A cross-sectional study

**DOI:** 10.1186/s12889-025-23772-3

**Published:** 2025-07-23

**Authors:** Yunes Nazzal, Faiz Dogru, Janna Schlenke, Fabian Holzgreve, Ioannis Karassavidis, Verena Komanek-Prinz, Rejane Golbach, Eileen M. Wanke, Gerhard Oremek, David A. Groneberg, Daniela Ohlendorf

**Affiliations:** 1 Institute of Occupational, Social and Environmental Medicine, Goethe University Frankfurt, Theodor-Stern-Kai 7, Frankfurt am Main, 60596 Germany; 2 Department of Health Care Management, Organizational Unit of the Federal Police, Berlin, Germany; 3 Clinic for Psychiatry, Psychosomatics and Psychotherapy, Goethe University Frankfurt, Theodor-Stern-Kai 7, 60596 Frankfurt am Main, Germany; 4 Institute of Biostatistics and Mathematical Modeling, University Hospital Frankfurt, Goethe University, Theodor-Stern-Kai 7, 60596 Frankfurt am Main, Germany

**Keywords:** Police officers, Germany, PSQ-Op, COPSOQ, Well-being, Work-related stress

## Abstract

**Background:**

Psychological and social health is particularly important for police officers to perform their duties. Therefore, the present study identified and analysed relevant influencing psychosocial factors on officers of a state police unit in Germany.

**Methods:**

253 (209 m/44f) police officers from a single, federal state police force in Germany volunteered to answer questionnaires which comprised the Copenhagen Psychosocial Questionnaire (COPSOQ) and the Operational Police Stress Questionnaire (PSQ-Op). In addition, based on the response behaviour to each question, an Item Response Theory (IRT; Rasch model) was applied. This calculated score (Rasch score) can be regarded as a predictor of well-being (COPSOQ) and work-related stress (PSQ-Op) and was correlated with age, body mass index (BMI), weight, height and years/hours of work. The significance level was set at 5%.

**Results:**

COPSOQ: police officers reported moderate to highlevels of emotional exhaustion (41.1%) despite moderate to balanced emotional workloads and the need to suppress emotions (38.33%). PSQ-Op: about one-third of the police officers found shift work moderately stressful (27.27%), while 22.55% reported work-related health problems. Both questionnaires revealed good collegial cooperation (> 90%) and satisfaction with this (> 90%).

**Rasch score:**

Rasch scores of the COPSOQ showed significant negative correlations with working hours (*p* = 0.001, rho = -0.16), higher body weight (*p* = 0.007, rho =-0.17) and higher body height (*p* = 0.003, rho = -0.19). A significant positive correlation was established between the officer´s age and their working years (*p* = 0.001, rho = 0.65). The Rasch scores of the PSQ-Op had a significant positive correlation with younger age (*p* = 0.001, rho = 0.21) and fewer working years (*p* = 0.03, rho = 0.13). No significant correlation was found between the Rasch scores of the PSQ-Op and COPSOQ.

**Conclusion:**

German police officers experienced moderate emotional strain accompanied by moderate emotional exhaustion, however, this had little influence on decision-making. Good collegial cooperation and low physical strain contributed to low-moderate stress levels. Overtime and working when ill may also be contributing factors for physical exhaustion. The majority of officers fulfilled their occupational duties with undiminished energy. Job-related factors, such as effective cooperation among colleagues, appeared to have a positive impact on mental health.

## Introduction

The police profession requires specific assessments to address the physical, mental and emotional demands since its officers frequently experience high stress levels during duties such as patrol work [1, 2]. Psychosocial factors such as stress, hostility or resilience in policing [3] impact the police officer´s performance and mental health, with negative factors potentially compromising decision-making in dangerous situations where the life of a civilian or their own life could be at risk [4]. Deschenes et al. [5] identified three main factors affecting police officers’ psychosocial well-being: socioeconomic, organizational and personal factors. Several approaches or scientific models [2, 6–10] have been developed in order to investigate the potential impact of demands on the psychosocial health of law enforcement workers.

Research using the Job Demand Control (JDC) model has demonstrated that low job control combined with high job demands increases stress and mental health risks [8, 9]. In Sweden, 73% of police officers were found to experience low job control, although 88% reportedly received strong colleague support [8], while 38% of Abu Dhabi police officers with negative perceptions of work and health management have reported increased early retirement intentions [7].

The Police Officer Stress Questionnaire (PSQ-Op; evolved and validated by McCreary et al. (2006) [11]) has proven invaluable for studying police stress. Here, 43% of Brazilian police officers reported stress symptoms (54% among women) [12], while Portuguese officers showed high levels of work-related (89%), operational (84.4%) and social (76.2%) stress with significant psychological exhaustion [13]. These findings demonstrate the urgent need for stress prevention measures.

Physical characteristics also significantly impact police performance. Henze et al. [14] demonstrated that taller officers generally coped more effectively with physical demands, while studies by Dawes et al. [15] demonstrated that overweight police officers often scored lower in defensive tactics training. This negative correlation between BMI and job performance is associated with reduced aerobic strength and muscular endurance [16]. Psychological factors are equally important, as higher psychological hardiness correlates with lower BMI and better stress resilience through improved control and reduced stress hormone release [17].

Although the PSQ-Op has been applied internationally among police officers [7, 10, 13, 18], it has not been applied to German police units, up to now. The Copenhagen Psychosocial Questionnaire (COPSOQ; German version validated by Nübling et al. [19]) has, so far, been used to investigate psychosocial factors across various medical professions [20–23]. Unlike the more narrowly focussed JDC model, the COPSOQ offers a broader and more nuanced assessment of psychosocial work factors by including dimensions such as influence, recognition, social support and work-life balance. For this reason, it was used for the first time for German police officers in this study instead of the JDC model. Therefore, this study has addressed this gap by investigating psychosocial workplace factors affecting mental well-being and work-related stress in German police officers. These aspects, amongst others such as musculoskeletal complaints and postural patterns, have been researched by our group in a larger project [24]. By combining these tools, we can examine psychosocial factors more comprehensively [7, 13, 21] than the JDC model alone, while also considering demographic data such as age, height, BMI, working years and overtime hours that may influence an officer’s well-being and performance.

Therefore, three hypotheses were tested:

1. Larger body size reduces physical strain in police work, while age, years of service and overtime increase work-related stress and exhaustion.
2. Emotional strain and exhaustion have no effects on the police officer’s decision-making.
3. Collegial cooperation positively impacts mental health and reduces stress among German police officers.

## Materials and methods

The study was conducted between July and October 2022.

### Subjects

Overall, the total cohort of 253 (209 m/44f) police officers from a single, federal state police force in Germany participated in this study. Three questionnaires were excluded from the analysis due to incomplete responses as the participants did not complete all items up to the final question. The included participants were between 21 and 57 years old (mean age 28.28 ± 6.55 years), had an average height of 179.76 ± 10.65 cm, an average weight of 81.9 ± 12.36 kg and an average BMI of 26.1 ± 2.37 kg/m^2^. Gender-specific characteristics are shown in Table 1.

**Table 1:** General characteristics (body height, body weight, BMI and age) of all the participants and when separated by sex (male and female Police officers)

	All subjects (*n* = 253)				Male police officers (*n* = 209)				Female police officers (*n* = 44)				
	Mean value	SD	Median	1st./3rd quartile	Mean value	SD	Median	1st/3rd quartile	Mean value	SD	Median	1st/3rd quartile	*p*-value
Body height (cm)	179.76	10.65	180.00	175.00/185.00	182.64	6.62	182.00	178.00/187.00	166.31	14.70	168.00	164.00/172.00	0.001
Body weight (kg)	83.5	28.89	83.00	74.00/90.00	87.57	30.08	85.00	80.00/91.00	63.91	5.85	63.00	59.00/69.50	0.001
BMI (kg/m^2^)	25.53	6.77	25.86	23.37/26.77	26.3	7.26	25.59	24.13/27.15	24	1.69	22.33	21.33/23.73	0.001
Age (years)	28.28	6.55	26.00	24.00/31.00	29.08	6.88	27.00	24.00/32.00	24.51	2.27	24.00	23.00/25.75	0.001

**Fig. 1:**
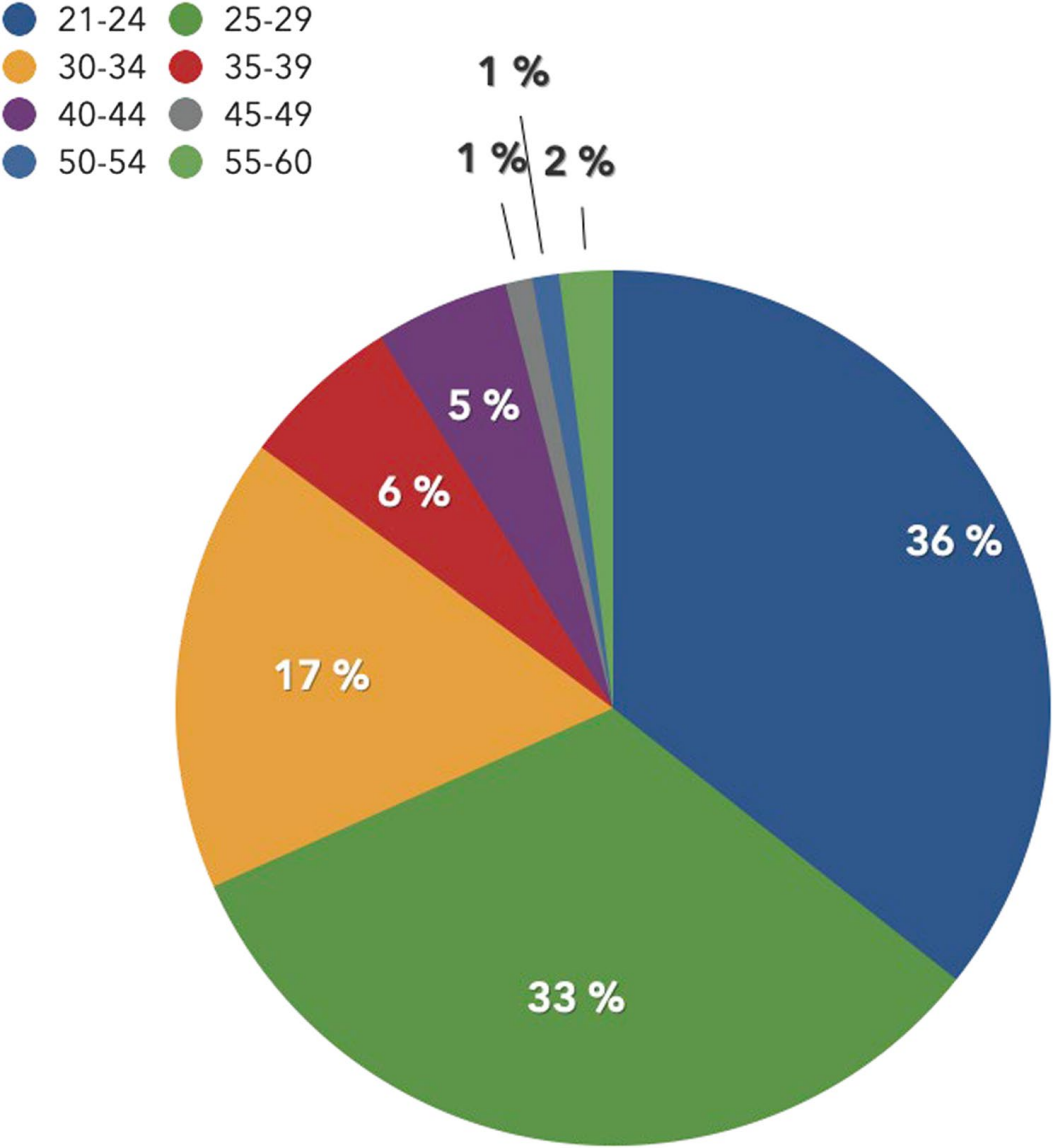
Distribution of the participants’ age ranges (in years) and the equivalent percentage of the sample size represented by a pie chart

**Fig. 2:**
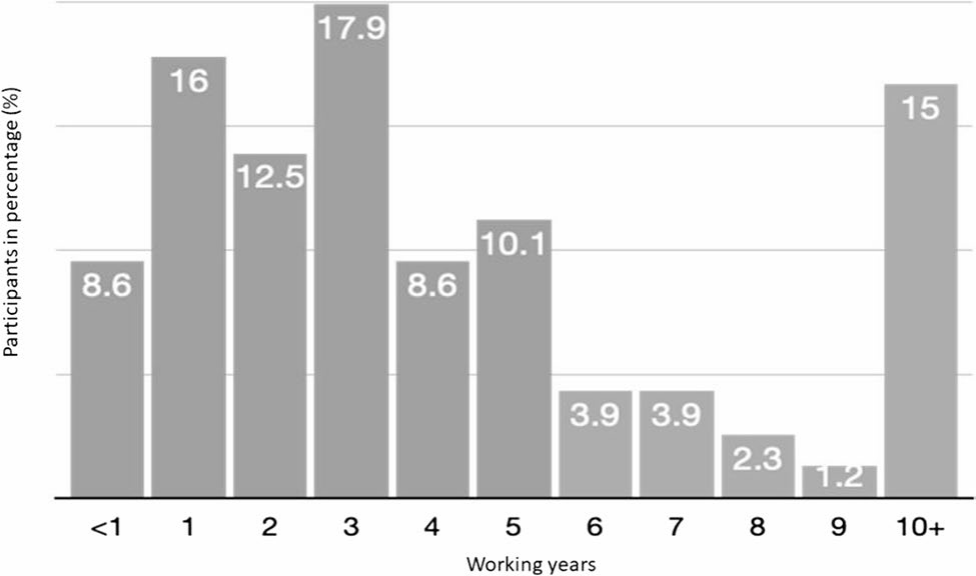
Distribution of the participants’ years of working for the German police with the equivalent share of the sample size in percent %

Furthermore, Figs. 1 and 2 depict the diverse distribution ranges concerning age and years of service. The biggest age group lay below 24 years (36.2%), followed by the second largest group the 25- to 29-years-olds group (32.9%) (Fig. 1). In addition, 55% of the participants had worked for the police for a maximum of three years (Fig. 2).

Inclusion criteria for the subjects were that they should be police officers working for the German police department and be on active duty. Exclusion criteria mainly involved police officers who did not participate in active duties, as well as officers who were working for countries other than Germany. We have submitted a study proposal with an approved ethics application from the Department of Psychology and Sports Science at the Goethe University Frankfurt am Main (No.: 2022-07).

### Recruitment

Prior to the survey, informational events were held in which the study and its scientific objectives were presented. All supervisors of the organisational units involved in the survey were present at these briefings. These supervisors subsequently communicated the relevant information to their respective colleagues. Participation in the survey was voluntary. There was a high level of interest among the staff, as they were eager to see the outcomes of a university-led study that directly addressed their specific concerns. Although participation was formally voluntary, the organisational culture within the units strongly encouraged participation, in line with the hierarchical structures typical of public sector authorities. Throughout the entire process, official administrative protocols were strictly followed.

By focussing on general objectives rather than specific outcomes during the pre-survey briefing, suitable conditions were established to minimise potential influences on the participants’ responses, thereby contributing to the robustness of the collected data. Further information can be found in Ohlendorf et al. [24].

### Questionnaire survey

Both questionnaires were uploaded to the online platform of the SoSci Survey server where the participants could access them by scanning a QR code. Only fully completed questionnaires were included in the statistical analysis: incomplete ones were excluded. Due to incomplete demographic information, three questionnaires had to be excluded from the analysis. A pretest was conducted with students from the German Police Academy to evaluate the comprehensibility, response consistency and potential biases of the questionnaire prior to its administration to the target group [24]. The selection and exclusion of specific items were systematically assessed to ensure the psychometric validity and reliability of the scales. Due to time constraints and the professional context of the sample group, the COPSOQ was abbreviated. The decision to remove certain items was based on both theoretical and methodological considerations, ensuring that the adapted versions of the COPSOQ and PSQ-Op retained their analytical robustness and relevance. All modifications to the survey instrument were thoroughly reviewed and approved by official representatives from the German Police Department.

### Copenhagen psychosocial questionnaire (COPSOQ)

This survey incorporates the German version of the COPSOQ, a self-evaluation tool to assess psychosocial elements in the work environment. The original questionnaire was presented successfully for the first time in 2005 by Kristensen et al. [25] when it was used to assess psychosocial factors at work, stress and the well-being of workers in the public field in Denmark via 141 questions [19]. An international validation study confirmed the validity and reliability of the questionnaire, independent of the language [11]. The COPSOQ is now used worldwide and has been translated into many languages (English, Polish, Spanish, Portuguese, etc.) [25–27]. Regarding the present study, a German version was used that had been validated in the study of Nübling et al. [19] in 2006. In this investigation, an occupation-specific modified version was used. A pretest was executed with *n* = 200 police officer students [24].

In this survey, we covered various aspects including the work demands, influence and growth prospects, work-related factors, social relationships and leadership. From these factors, we were able to assess the possible relationships and impacts on job satisfaction, engagement and overall health.

**Fig. 3:**
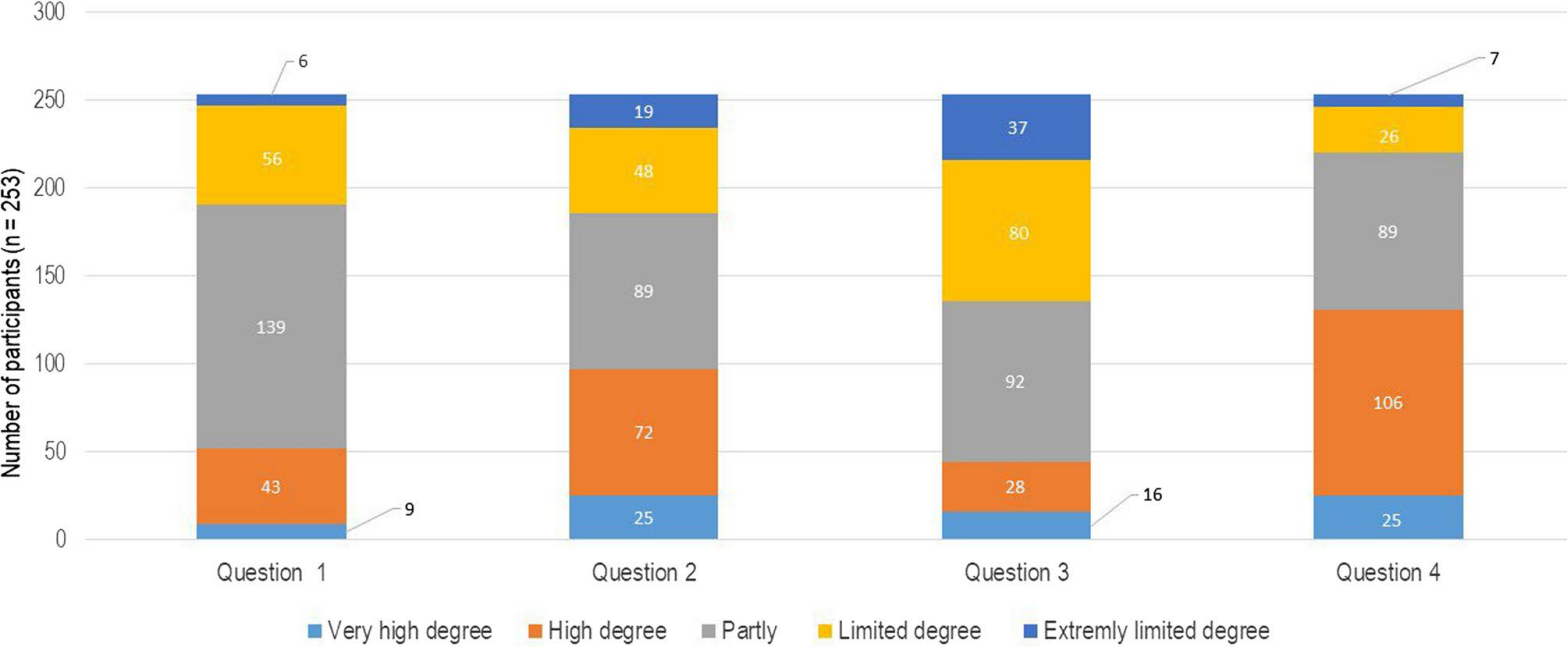
Graphical representation of the answers for questions on emotions from the COPSOQ. The numbers on the x-axis represent the questions: (1) Does your work entail a significant emotional demand? (2) Does your work require you to hide your own emotions? (3) Does your work demand so much energy that it negatively impacts your personal life? (4) Are you able to apply your skills to your job? The y-axis demonstrates the distribution of the participants (n)

**Fig. 4:**
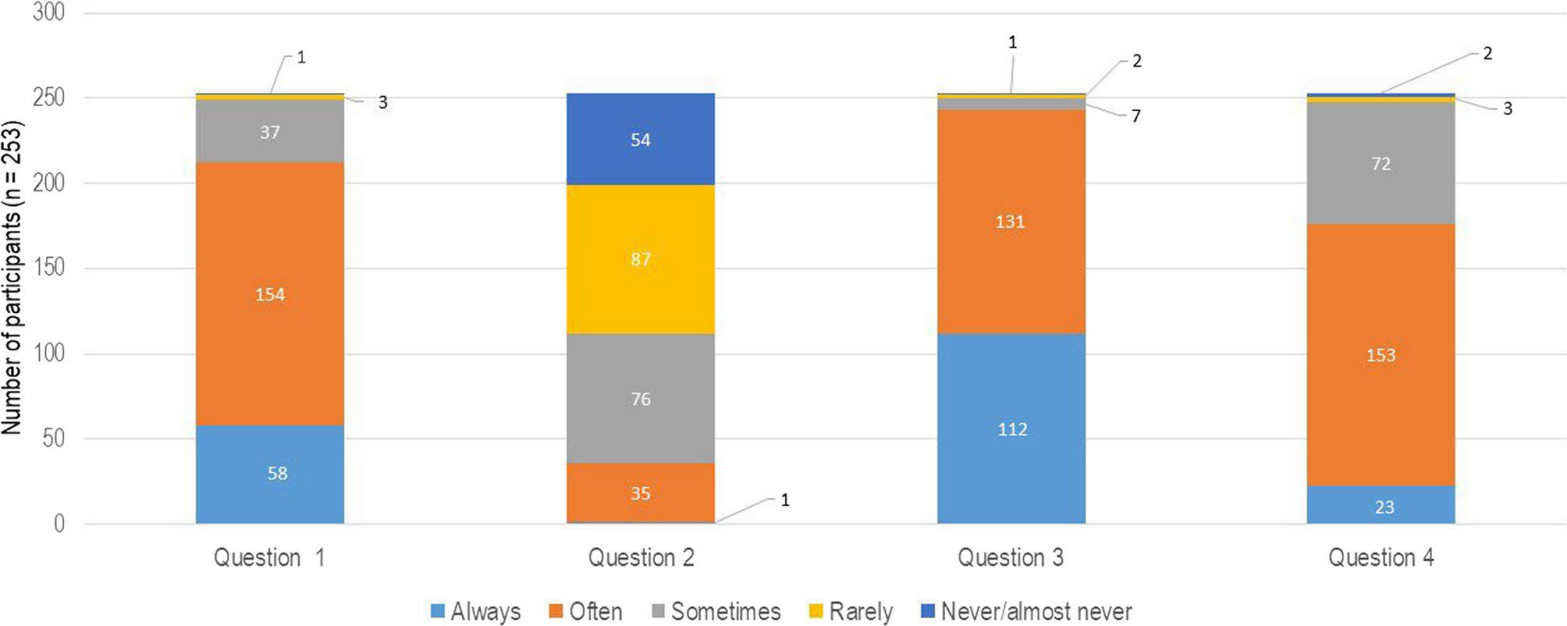
Graphical representation of the answers to the question concerning quitting their occupation taken from the COPSOQ. The x-axis represents the question 1. How often have you considered leaving your occupation in the past 12 months? The y-axis represents the distribution of participants (n)

**Fig. 5:**
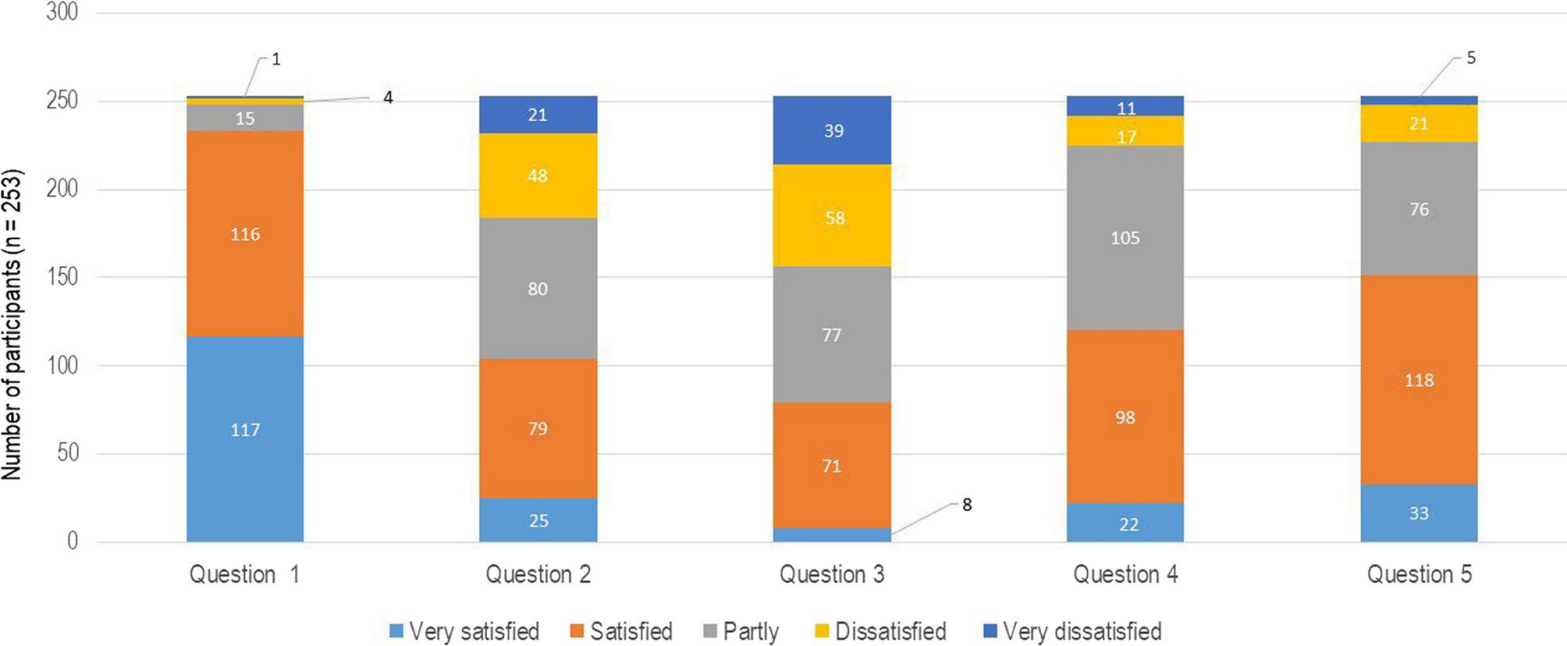
Graphical representation of the results from the Operational Police Stress Questionnaire. This questionnaire describes the following aspects of police work (x-axis): (1) Shift work; (2) Risk of injuries during work; (3) Paperwork; (4) Having enough time to keep yourself in a good physical condition; (5) Occupational health issues. The colour coding of responses 1–7 on the y-axis describes the intensity of stress levels from dark blue (1: no stress at all) through to orange (4: moderate stress) to medium blue (7: a lot of stress); the higher the number of the colour, the greater the stress level

**Fig. 6:**
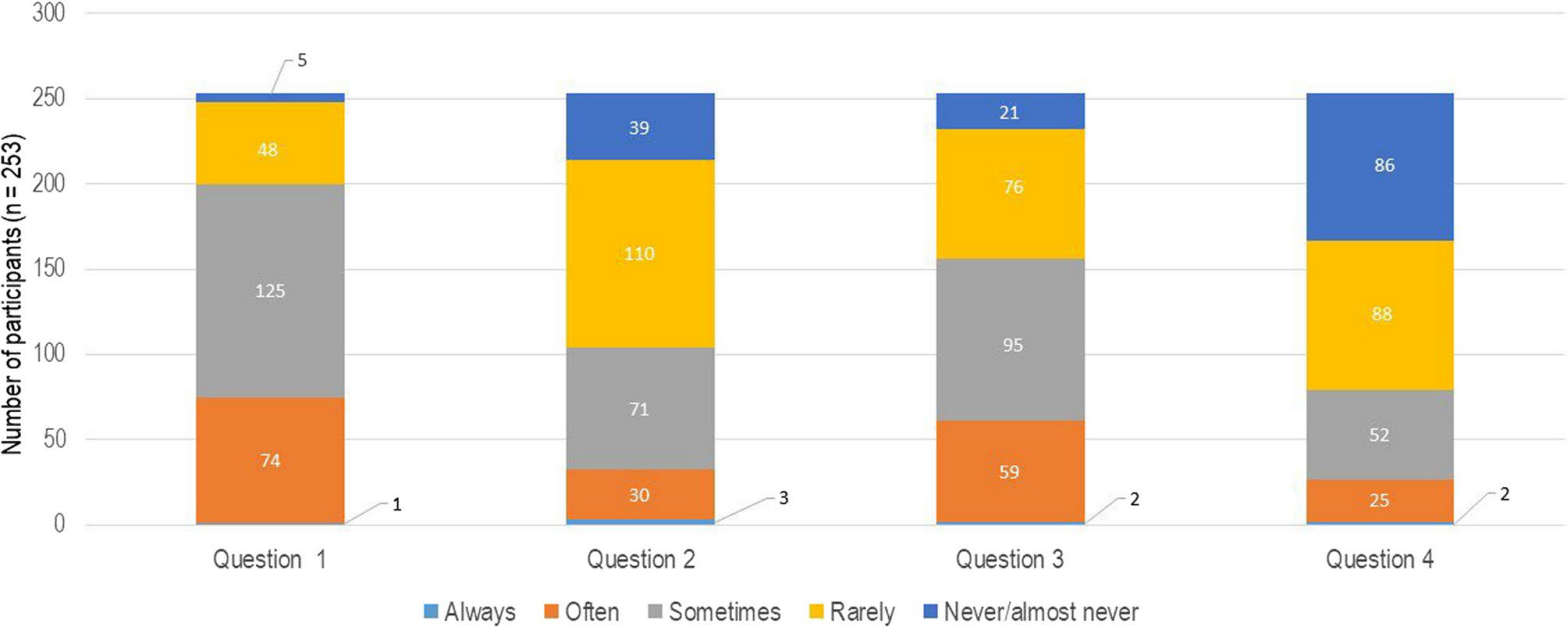
Graphical representation of the answers for questions concerning the working environment from the COPSOQ. The numbers on the x-axis represent the questions: (1) Do you have to work overtime? (2) Do you have a significant influence on decisions concerning your work? (3) Is the cooperation with colleagues good? (4) How far does the following statement apply: I am full of energy at work? The y-axis demonstrates the distribution of participants (n)

**Fig. 7:**
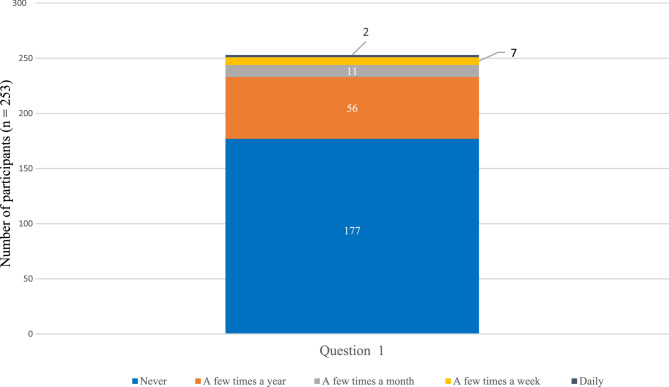
Graphical representation of the answers to questions concerning the physical state from the COPSOQ. The numbers on the x-axis represent the questions: (1) Are you physically exhausted? (2) Are you emotionally exhausted? (3) Are you feeling drained? (4) Do you come to work even when you are feeling sick/unwell? The y-axis demonstrates the distribution of participants (n)

The answer options for the subjects from the COPSOQ ranged from “to a very high extent” to “to a very low extent”, “always” to “never/almost never”, “very satisfied” to “very dissatisfied” and a timely answer from “never” to “few times a year” to “daily”. Participants of the study could only choose one out of the five possible answers and every single question was evaluated independently. All questions that were relevant for the present study are shown in Figs. 3, 4, 5, 6 and 7.

### Operational police stress questionnaire (PSQ-Op)

The PSQ-Op was devised in 2006 by McCreary and Thompson [11] and has been used as a valid and reliable assessment [18, 19] since 2013. This questionnaire aimed to investigate the possible relationships between physical health, stress and psychological well-being that are specific to police officers [28, 29]. The German version offered, as answer possibilities, a numerical rating scale ranging from 1, which was defined as moderate low stress, up to 7, which identified a high level of stress (0–3 means no/low stress, 4–5 a moderate level of stress and 6–7 high levels of stress). Each question was individually evaluated.

**Fig. 8:**
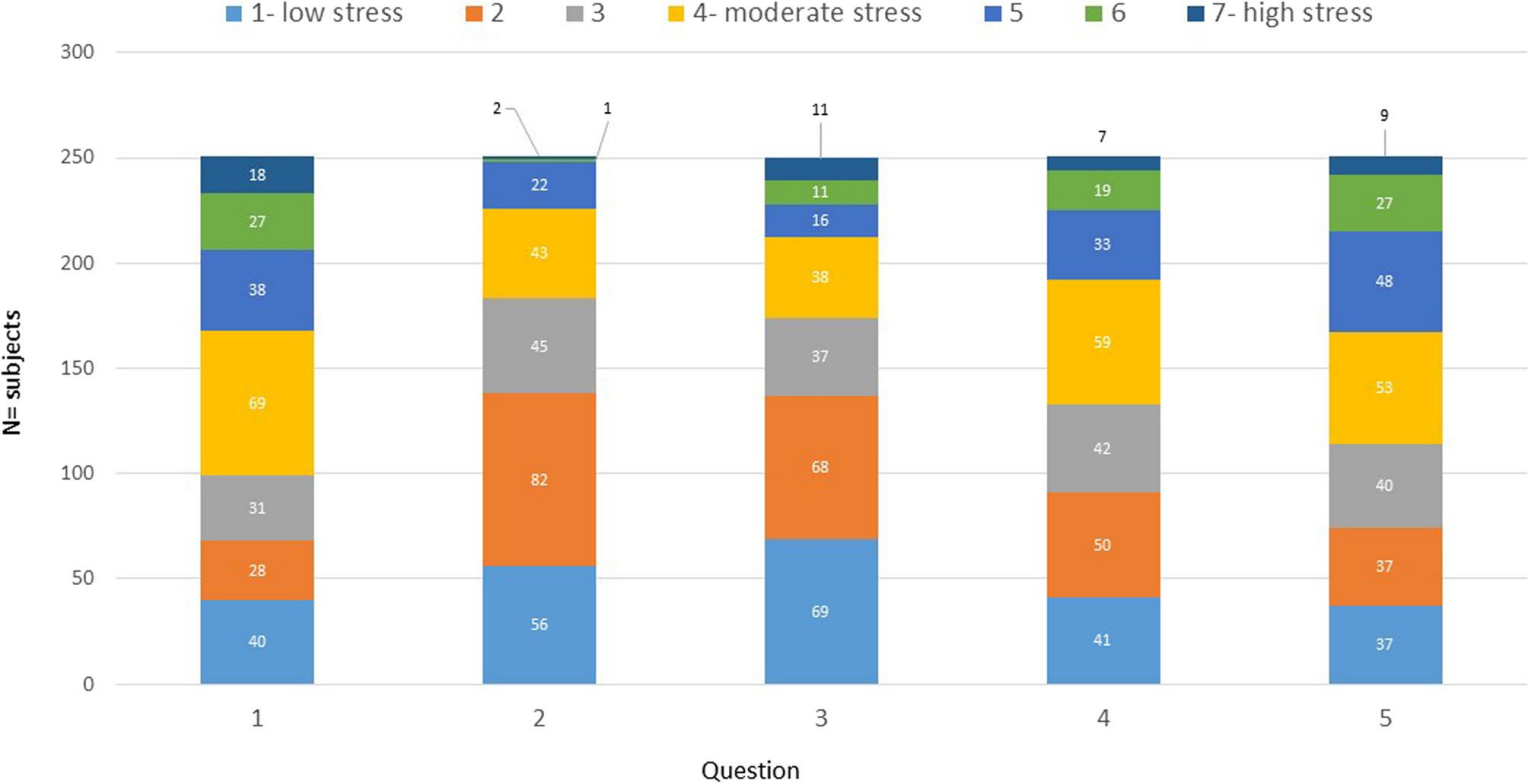
Graphical representation of the answers to questions concerning the departmental environment from the COPSOQ. The x-axis is represented by the questions on satisfaction:1…with people you work with?2…with how the department is managed/organised? 3…with the wage/salary 4…with the physical conditions 5…with the occupational opportunities. The y-axis represents the distribution of participants (n)

Participants were instructed to select only one response out of seven answer options per question. In the present study, the original 20-item questionnaire was condensed to five occupation-specific questions (Fig. 8) in order to align more strongly with the unique demands and context of police work.

Demographic data, including age, body weight, height and body mass index (BMI), were collected at the beginning of the questionnaire and completed by all study participants. Participants were instructed to provide responses to the best of their knowledge and ability to ensure the accuracy and reliability of the data.

### Statistical evaluation

Data collection and descriptive evaluation of the data, including the generation of graphs and the calculation of absolute and relative frequencies, were performed in Excel. Rasch scores based on the polytomous Rasch model by Nübling et al. and Tennant and Conaghan [19, 30] were computed for the set of selected items from the COPSOQ questionnaire, and separately for the set of selected items from the PSQ-Op questionnaire, in R (version 4.3.3) with the “TAM” package. Further analyses were performed with the programme SPSS Statistics 26 (IBM Deutschland GMbH, Ehningen, Germany).

Since no validated scores exist for the sets of selected items and the study population, overall scores provided by the polytomous Rasch model were used to determine a relative assessment of work-related well-being (based on COPSOQ) and work-related stress (PSQ-Op) for each participant. The focus of the Rasch analyses was not on evaluating the performance of individual items but rather on deriving a score for each subset of questions. For both derived scores, higher values indicated a more negative outcome, i.e. higher scores for work-related well-being reflected a lower well-being, and higher scores for work-related stress corresponded to greater stress.

The Wilcoxon-Mann–Whitney U test was conducted to assess differences in anthropometric parameters between male and female police officers. For testing correlations between the participant’s characteristics (such as age, body weight, body height, body mass index, working years, working hours) and work-related well-being and stress, respectively, the Spearman’s rank correlation coefficient was calculated as the Rasch scores were not normally distributed. Normal distribution of the Rasch scores was tested using the Kolmogorov-Smirnov-Lilliefors test. For the interpretation of the magnitude of absolute correlations, the interpretation according to Evans [31] was applied and showed that values of < 0.2 were considered poor, 0.2–0.4 weak, 0.4–0.6 moderate, 0.6–0.8 strong and > 0.8 optimal. The significance level was set at 5%.

## Results

### Copenhagen psychological questionnaire (COPSOQ)

In Figs. 3 and 52 (20.55%) participants answered that their work entailed an important emotional demand (question 1), while question 2 revealed that 97 (38.33%) had to hide their own emotions during work. On the other hand, 117 (46.24%) participants answered that the work demands did not have a negative impact on their private lives (question 3). In question 4, 131 (51.77%) police officers answered that they were able to apply their skills to their job.

Figure 4 presents a graphical representation of the answers to questions concerning overtime. Question 1 revealed that 212 (83.79%) officers often needed to work overtime, whilst question 2 illustrated that 141 (55.73%) of the participants confirmed that they had a little influence in decision-making at work. Another important result is the 3rd question of Fig. 4, regarding the cooperation between colleagues; in this case, a major group of 243 officers (96.04%) confirmed a good cooperation between colleagues. Represented in response to question 4, 176 (69.56%) police officers expressed that they had the feeling that they were full of energy while being at work.

Figure 5 shows the individual’s experience of working for the German police, highlighting how they were personally affected and how they perceived the work demands from their own perspective. In this context, 75 (29.64%) police officers felt physically exhausted (question 1), while 33 (13.04%) participants felt emotionally exhausted (question 2). Sixty-one officers (24.11%, question 3) felt drained by their work demands, while 174 (68.77%) officers rarely showed up for work, or not at all, when they had the feeling that they were sick or unwell, as revealed by question 4.

Furthermore, the questionnaire contained questions relating to the satisfaction of the officers regarding their work (Fig. 6). Of the study sample, 233 (92.09%) were satisfied/very satisfied with the people that they were working with. Question 2 revealed that 104 (41.10%) officers were satisfied with the organization and management of their department, while question 3 confirmed that 97 (38.33%) of the participants were not satisfied with their wage/salary for working in the German police. However, the results from question 4 showed that 120 (47.43%) officers were satisfied with their physical conditions at work. The last question (question 5) revealed that 151 (59.68%) participants were satisfied with their occupational opportunities.

A large group of 177 (69.96%) of the participants had never thought, in the past 12 months, of leaving their occupation, while only 2 (0.79%) of the officers were thinking daily of leaving their occupation (Fig. 7).

Work-related well-being (Rasch Score COPSOQ) was correlated with working hours, body weight, body height, age, the BMI and the number of working years. A negative correlation was found between work-related well-being and body weight (*p* = 0.01, rho = −0.17), as well as body height (*p* = 0.003, rho = −0.19). No significant correlations were observed with age, the BMI or the number of working years.

### Operational Police stress questionnaire (PSQ-Op)

The participants answered, using a scale (Fig. 8), on how stressed they were in the past six months by regarding a couple of keywords. The scale ranged from 1, which was defined as low stress, up to 7, which identified a high level of stress. Figure 8 shows the different distribution of the participants’ answers in numbers: 69 (27.27%) of the participants related a moderate stress level of 4 out of 7 when being confronted with the term ‘shift work’, as seen by question 1. Regarding ‘risk of injuries at work’, the second question highlighted that 82 (32.41%) of the officers answered to have had a low stress level of 2 out of 7. Furthermore, question 3 showed that 69 (27.27%) of the sample size showed a very low level of stress when thinking about the key word ‘paperwork’. Regarding question 4 concerning proper time management, in order to ‘have enough time to keep yourself in a good physical condition’, a majority of 59 (23.32%) officers showed a moderate stress level of 4 out of 7. An similar result was established concerning the keywords ‘occupational health issues’ in question 5, where 53 (22.55%) of the officers answered as having the same stress levels in response to the last question.

Since police officers are sometimes involved in certain operations, they may have to work overtime in the working week. Among the 76 supervisors, 47.14% experienced 1–4 h per week overtime work, while 31.43% were working 5–9 h per week overtime. However, 17.15% were not working overtime or worked less than 41 h per week. The remaining 4.28% were working more than 11 h of overtime per week. Among the 177 non-supervisors, 38.82% experienced 1–4 h per week overtime work, while 27.06% were not having any overtime hours per week and 4.11% were working for less than 41 h per week. For the officers, 5–9 h overtime was found within 24.12% of the sample, while over 11 h overtime per week was acknowledged by 5.88% of the officers.

Work-related stress (Rasch Score PSQ-Op) was correlated with age, working years, body height, the BMI and body weight. A positive correlation was found between work-related stress and age (*p* = 0.001, rho = 0.21), as well as working years (*p* = 0.003, rho = 0.19). No significant correlations were observed with body height, the BMI or body weight.

Differentiation between the genders could not be made since there was not a notable difference in the results.

## Discussion

The aim of the present study was to assess the psychosocial factors that affect police officers´ work and their health outcomes. Our study on German police officers revealed a complex work environment where 20.6% reported significant emotional demands and 38.3% had to hide emotions at work; nevertheless, 46.2% stated that work did not negatively impact their private lives and 51.8% could apply their skills effectively. Overtime was common (83.8% of officers), with supervisors working more extra hours than non-supervisors. Despite limited decision-making influences (55.7%), workplace cooperation was exceptionally strong (96.0%) and 69.6% felt energetic at work. Physical exhaustion affected 29.6% of officers, emotional exhaustion 13.0%, and 24.1% felt drained by work demands, while 68.8% avoided working when sick. Job satisfaction was high with regard to colleagues (92.1%), moderate for department management (41.1%) and physical conditions (47.4%), although 38.3% were dissatisfied with their salary; nevertheless, 70.0% had never considered leaving their profession in the past year. Stress levels varied by stressor: shift work and time management for physical fitness caused moderate stress, while paperwork and injury risks generated lower stress levels with no notable gender differences observed in the results. Work-related well-being negatively correlated with body weight and height, while work-related stress positively correlated with age and years of service, suggesting that stress increases with career progression. These results confirmed hypothesis 1 that overtime demonstrably increases work-related stress and exhaustion, while collegial cooperation acts as a protective factor. However, the assumption about the relationship between emotional workload, limited decision influence and job dissatisfaction had to be falsified (hypothesis 2). The high levels of colleague satisfaction and satisfaction with teamwork (> 90%) observed in our study support this finding. Consequently, hypothesis 3 could be verified.

Comparing our results with those of international studies reveals both similarities and notable differences in the results. High emotional work strain and limited influence on decision making was found not only to lead to job dissatisfaction or health impairments among the respondents, but, in the long term, to increase the risk of burnout [9, 20]. The work of Heimann et al. [32] confirmed that, due to the different hierarchies within the police force, officers have a limited influence over their own decisions (55.73% of respondents) and are subject to the instructions of their superiors [32, 33]. Job-related factors such as good relationships with colleagues and good teamwork may have positive effects on mental health and explain the low levels of physical and emotional exhaustion [10, 34]. In general, satisfaction regarding the work demands and psychosocial factors among police officers aligns with the international literature [6, 10, 12, 34].

Although our surveyed German police officers reported only low emotional (13.05%) and physical (29.65%) overload, Riedy et al. [35] found that overtime negatively affected psychosocial factors and this led to psychological stress among US police officers. Similarly, Phadke et al. [33] documented high stress levels (59.75%) among Indian traffic police officers due to overtime, leading to increased behavioural stress. This finding was also supported by Violanti et al. [36], highlighting that overtime work and shift work are factors for occupational stress. It is also worth noting the contrast of the findings by Lipp et al. [12] from Brazil, where higher-ranking police officers showed a stress level of 7.8 out of 10, with the present study where 72.2% of the surveyed supervisors experienced little to no emotional exhaustion. These differences highlight the importance of cultural and organisational contexts when assessing work-related stress in the police service. The findings on collegial cooperation and its positive effects on mental health have been confirmed not only by the present study but also by several international studies: Swedish police officers have a significant relationship between the availability of social support or rather collegial cooperation and mental health [34]. Similarly, Andersson et al. [10] found that almost three-quarters of Swedish police officers were exhausted after work that was attributed to high job demands and low social support and, furthermore, they noted that increased support at work enhanced job satisfaction.

The findings of the applied analyses regarding the well-being and work-related stress (Rasch score COPSOQ), showed that police officers with higher stature and higher body weight rated questions about work-related well-being more positively. These results are in line with the assumption that the physical demands of wearing protective gear (body-, arm- and leg-protectors and helmets) for several hours [24], seem less taxing for larger or heavier officers when having to wear the gear, for example, during demonstrations or other situations when on duty. Due to the low effect size of these significant correlations, they are more likely to be a trend and have no clinical relevance.

Comparing our results with those of other occupational groups with similar stress profiles reveals interesting parallels and differences. Wagner et al. [37] identified high quantitative demands and difficulties in achieving a work-life balance in the healthcare sector as key factors for developing psychological strain, emphasising the importance of effective and structured leadership. De Jonge et al. [38] found even worse values for physical and psychological stress among 541 respondents from the healthcare sector in the Netherlands, thus indicating a possible counterproductive approach in this work area. In the Netherlands, the workers seemed to focus exclusively on their work, often thinking excessively about it and allowing themselves too little recovery time, and so negatively impacting their personal lives. Firefighters showed higher rates of overweight and obesity as well as significantly elevated occupational stress levels [39], while police officers surveyed in the present study generally reported high energy levels and low physical exhaustion. These cross-occupational comparisons underscore the importance of regular physical exercise and psychological support structures for maintaining occupational health.

However, the present study has several methodological limitations that should be considered when interpreting the results. Firstly, the cross-sectional design does not allow causal conclusions or longitudinal observations. Due to the pseudonymisation of the data, re-evaluation with the same police officers is not possible, thus preventing the tracking of changes over time. Another limiting factor is the unbalanced gender ratio favouring male participants, which meant a gender-specific analysis is not possible. Although this imbalance reflects the actual gender distribution in the German police service (71.7% men versus 21.3% women), it limits the possibility of investigating gender-specific differences in psychosocial stress. A balanced gender ratio would have limited the external validity [40]. Furthermore it should be noted that the inclusion criteria “less than one working year” refers to the practical year before applying to the federal police organisational unit and not to the subject’s entire practical work experience; therefore, this should be considered when interpreting the results for less experienced officers.

The strengths of the study include the use of standardised questionnaires such as the COPSOQ, which allow for the comparison of psychosocial well-being profiles between different occupational groups such as law enforcement, firefighting or healthcare [37, 39]. These occupations share characteristics such as physically and emotionally demanding work and carry a risk for psychological strain. Through pre-survey information about the scientific benefits of the study and the importance of truthful responses, an attempt was made to minimise response bias. Thus, questionnaires continue to remain the preferred method for quantitatively examining psychosocial stress factors [10, 34]. The clear responses to the PSQ-Op questions indicate the high validity of the results since the police officers took clear positions in their responses. Due to the conceptual overlap between the Job Demands-Resources (JDR) model and the Effort-Reward Imbalance (ERI) model, as well as the potential for increased complexity and redundancy in their interpretation, this study specifically utilises the Copenhagen Psychosocial Questionnaire (COPSOQ) and the Police Stress Questionnaire (PSQ-Op) to provide a more tailored and comprehensive assessment of the psychosocial factors affecting police officers. Not least, the study provides valuable insights into a previously little-investigated context through its focus on German police officers, since the COPSOQ and OSQ-OP had not previously been used for German police officers despite their international application.

Based on the results of this study, several practical implications for improving the psychosocial well-being and job-related stress of police officers can be derived. In order to maintain psychosocial well-being, measures should focus on strengthening team cohesion by maintaining and promoting further regular team meetings that encourage collegial exchange and mutual support. Improving collegial exchange would further strengthen the high level of collegial support and may, indeed, lower emotional exhaustion. Structured reflection sessions and emotional debriefings after stressful events should complement these efforts to support the feeling of suppression and the influence of one’s emotions during work and decisions. In addition, implementing peer support programmes, offering resilience-building interventions and training in emotional regulation strategies, as well as introducing flexible rotation models for emotionally and physically demanding tasks, may help mitigate emotional work strain and physical exhaustion. Promoting a participative leadership style to increase the officers’ influence on operational and organisational decisions, together with stress reduction initiatives and a health-oriented leadership approach, could be key to addressing effectively the psychological and physical demands placed on police officers. In addition, work-life balance plays an important role not only in maintaining work motivation but also in supporting mental health [41]. Therefore, a strict separation between work and leisure is essential and is important to ensure both physical and psychological recovery from stress [41].

Finally, preventive measures to promote physical fitness and mental well-being should be implemented [42]. As the study found a connection between height, body weight and a more positive assessment of work-related well-being, specific training programmes could be developed that are tailored to the physical demands of the police service, findings that reinforce the critical role of regular physical exercise and psychological support structures in maintaining occupational health [39].

These could facilitate the wearing of protective equipment over extended periods and, thus, reduce physical strain. Complementary psychological support services should be expanded to help police officers deal with emotional burdens and strengthen their resilience.

## Conclusion

This study examined psychosocial factors affecting officers in a German state police unit. The COPSOQ and PSQ-Op results revealed moderate emotional work demands with low emotional exhaustion despite limited decision-making autonomy and emotion suppression requirements. Good collegial cooperation and low physical strain contributed to moderate stress levels. While overtime and presenteeism caused moderate to high physical exhaustion for one-third of the officers, most reported high energy at work. We recommend a multifaceted approach to sustaining psychosocial well-being by enhancing team cohesion through regular meetings, structured emotional debriefings, peer support and resilience programmes, flexible task rotations and health-oriented leadership to sustain the officers’ psychosocial well-being.

## Data Availability

All data generated or analysed during this study are included in this published article.
